# Cardiac natriuretic peptides in plasma increase after dietary induced weight loss in obesity

**DOI:** 10.1186/s40608-014-0024-2

**Published:** 2014-12-02

**Authors:** Caroline Kistorp, Henning Bliddal, Jens P Goetze, Robin Christensen, Jens Faber

**Affiliations:** Department of Endocrinology, Medicine O, Endocrine Unit, Herlev University Hospital, Herlev Ringvej 75, Herlev, DK-2730 Denmark; The Parker Institute, Department of Rheumatology, Copenhagen University Hospital, Copenhagen, Denmark; Department of Clinical Biochemistry, Copenhagen University Hospital, Rigshospitalet, Copenhagen, Denmark; Faculty of Health Sciences, University of Copenhagen, Copenhagen, Denmark

**Keywords:** Natriuretic peptides, Body Composition, Hypo-caloric diet, Weight Loss, Obesity

## Abstract

**Background:**

Cardiac natriuretic peptides are established biomarkers in heart disease, but are also affected by body mass index (BMI). The purpose of the present study was to examine the impact of weight loss and changes in body composition following dietary intervention on plasma concentrations of the prohormones to A- and B-type natriuretic peptides (proANP and proBNP) and adrenomedullin (proADM).

**Results:**

A total of 52 healthy obese subjects, 47 women and 5 men (BMI 36.5 ± 5.6 kg/m^2^) were randomised to either an intensive weight reduction programme using a combination of very low calorie diet (810 kcal/day) and conventional hypo-energetic diet (1200 kcal/day) for 52 weeks, or to a control group that was offered diet-related counselling. N-terminal proBNP (NT-proBNP), mid-regional proANP (MR-proANP) and proADM (MR-proADM) and body composition using dual-energy x-ray absorptiometry (DEXA) scanning were determined at baseline and after 52 weeks. Comparisons between groups were analysed using t-tests. Changes from the baseline within the groups were analysed with paired tests. Changes in the variables, delta (∆), were calculated as 52 weeks minus the baseline.

In the intervention group, BMI decreased by almost 20% (31.6 ± 6.2 vs. 37.1 ± 6.1 kg/m^2^; P <0.001) with a loss of body fat of 23.5 ± 15.5% (P < 0.001). Plasma concentrations of NT-proBNP and MR-proANP increased (from 55 ± 31 to 97 ± 55 pg/ml; P < 0.001, and from 59 ± 21 to 74 ± 26 pmol/L; P < 0.001), whereas MR-proADM decreased (from 573 ± 153 to 534 ± 173 pmol/L; P <0.001). Changes (Δ) in MR-proANP correlated with Δfat mass (r = −0.359; P = 0.011) and Δglucose (r = −0.495; P <0.001), while increases in NT-proBNP were primarily associated with reduced plasma glucose (r = −0.462; P <0.001). A modest but significant weight loss of 6% (P < 0.001) was found in the control group with no changes in plasma concentrations of NT-proBNP or MR-proANP, and a minor change in MR-proADM.

**Conclusions:**

Plasma NT-proBNP and MR-proANP concentrations increase and MR-proADM concentration decreases during weight loss, underlining the dynamic impact of BMI, body composition and glucose metabolism on these cardiovascular biomarkers.

## Background

Obesity is an established risk factor for heart disease. However, cross-sectional studies have demonstrated that plasma concentrations of the cardiac B-type natriuretic peptide (BNP) including the N-amino terminal part of the prohormone (NT-proBNP) are decreased in obese subjects, being inversely associated with body mass index (BMI) [1]. The A-type natriuretic peptide (ANP) and BNP are normally synthesised in the cardiac atria and ventricle and secreted in response to wall-stress and neurohumoral stimulation. Markedly increased plasma concentrations are mostly found in patients with heart failure. Accordingly, cardiac natriuretic peptides (NPs) and their prohormones are now established as useful diagnostic biomarkers of heart failure [2], and they possess prognostic information both in healthy subjects and in patients with cardiovascular disease [3,4]. The diagnostic sensitivity of NT-proBNP and BNP could be affected in obesity, whereas ANP may be less influenced by BMI [5]. The mechanism leading to low plasma BNP and NT-proBNP concentrations in obesity is still not clarified. A clearance receptor for BNP (but not for NT-proBNP) has been identified in adipocytes, which suggests a possible role for both adipose and muscle tissue in altered clearance and degradation of the peptides [6].

With regard to the impact of weight loss in humans, available data from observational studies provide conflicting results [7,8]. Therefore, whether changes in body weight and in body composition following dietary intervention have a clinically significant impact on A and B-type NP concentrations in plasma is not resolved. Measurements of whole body composition, as assessed by dual-energy x-ray absorptiometry (DEXA), may provide novel information on the relationship between NPs in plasma and BMI.

Adrenomedullin (ADM) is a vasoactive peptide expressed in various tissues including adipocytes and vascular endothelium [9]. Circulating ADM has a short half-life but can be indirectly estimated by measurement of the mid-regional part of the prohormone (MR-proADM), which is more stable in plasma [10]. Recently, MR-proADM has been suggested as a promising novel biomarker for cardiovascular disease [11]. The expression of ADM is upregulated in adipose tissue in obese [12], but studies in clinical settings have reported inconsistent data in association with BMI. In patients with type 2 diabetes mellitus (DM) and with heart failure no association with BMI has been observed [5,13]. In contrast, a positive and independent association with BMI in severely obese subjects has been reported [14]. To our knowledge, previous studies of the impact of BMI on MR-proADM concentrations have not evaluated body composition as assessed by DEXA scan. Furthermore, the effect of weight loss after dietary intervention on circulating levels of MR-proADM is not known.

To examine the impact of changes in weight and body composition, we performed a randomised controlled prospective trial assessing the consequences of weight loss on plasma concentrations of MR-proANP, NT-proBNP and MR-proADM in obese individuals without heart disease.

## Methods

### Participants and design

Patients were enlisted at the outpatient clinic of the Parker Institute, Frederiksberg University Hospital, Denmark [15]. The primary inclusion criteria was BMI >28 kg/m^2^, and a clear motivation for participating in an intensive weight loss programme. Subjects were excluded according to the following criteria: known heart disease, DM by history or fasting plasma glucose above 7.0 mmol/L, known endocrine dysfunction, thyroid stimulating hormone (TSH) outside normal range, or impaired renal function. All the participants had s-creatinie within the normal range of our laboratory, maximum level being 102 μmol/l.

Screening blood measurements included: fasting plasma glucose, haemoglobin, serum-creatinine, liver enzymes and TSH. In addition, fasting lipids measurements were obtained. The study was approved by the Copenhagen ethical committee (ID: KF 01-104/02), and all subjects provided written informed consent. The design of this randomised controlled trial has recently been described in detail. Briefly, a total of 96 participants with knee osteoarthritis were randomised [15]. The primary reason for drop out from the study was lack of continued motivation, lack of compliance and knee surgery. In the present biomarker sub-study, participants were included according to the following criteria: completion of the baseline visit and the final visit after 52 weeks, including measurements of biomarkers and body composition by DEXA scan. The inclusion criteria of the biomarker study were pre-specified and randomisation status and parameters evaluated in the study were blinded from the technician analysing the biomarkers. Measurements of biomarkers and DEXA scan at baseline and after 52 weeks were available from 62 participants. Of these, 10 were excluded from the present analysis due to elevated plasma NT-proBNP concentrations at baseline >125 pg/ml, which is the recommended cut-off value to rule out a diagnosis of heart failure [16]. Therefore, in the present sub-study of our previously published randomised trial [15], we investigated 52 obese participants, 28 randomized to the intervention and 24 to control group.

### Dietary intervention and measurements

The intervention consisted of the following 52 weeks of intensive weight reduction programme: an initial 8 weeks of very low-calorie diet (VLCD) of 810 kcal/day, followed by 24 weeks of a hypo-energetic high protein diet of approximately 1200 kcal/day with guidance through weekly group sessions, followed by another 4 weeks of VLCD and finally 16 weeks where the participants attended group sessions every second week with the same experienced dietician. The VLCD consisted of nutrition powder (Speasy®, Dansk Droge A/S), which met the recommendations of a daily intake of high quality protein; 37 energy percent from protein, 47 energy percent from carbohydrate and 16 energy percent from vegetable fat.

Participants randomised to the control group attended a presentation by the same dietician treating the VLCD group and were recommended a diet that would provide them with approximately 1200 kcal/day. The participants were examined at baseline and after 52 weeks. Body weight was measured on a decimal scale (TANITA BWB-600S, Copenhagen, Denmark), body composition was measured using whole body DEXA scan (Norland DXA XR-36) measuring total body mass divided into three compartments of: bone, fat mass and lean mass. DEXA is a well established method of assessing body composition and has been validated previously in obesity research [17]. The same machine was used for all the measurements to minimise interscan variability. Venous blood was collected into standard tubes containing EDTA, promptly centrifuged at 4°C, and frozen at −80°C until analysis. The estimated glomerular filtration rate (eGFR) was calculated according to the MDRD formula. NT-proBNP levels were measured using a double antibody sandwich assay (Elecsys 2010, Roche Diagnostics). The lower detection limit is 5 pg/mL, and the intra-assay coefficient of variation (CV) was <2.7%, with a total precision CV of 5% [18]. MR-proANP and MR-proADM levels were measured with an automated sandwich immunoassay on the KRYPTOR system (BRAMS AG, Germany) at the Department of Clinical Chemistry, Rigshospitalet. For MR-proANP, the lower detection limit was 4.5 pmol/L and the intra-assay CV was 1.2%, with a total CV of 5.4%. For MR-proADM, the lower detection limit was 0.23 nmol/L, intra-assay CV 1.9%, and the inter-assay CV was 10% [10].

### Statistical analysis

Data are presented as mean ± SD unless stated otherwise. Comparisons between the intervention and the control group were analysed using independent *t*-test, or the Chi-square test for categorical variables. Changes from the baseline within the groups were analysed with paired tests. Changes of variables during the study period, delta (∆), were calculated as 52 week minus the baseline. Mean differences in ∆ values between the control and the intervention groups and 95% confidence interval (CI) were calculated by independent two-sample *t*-test. Predictors of NT-proBNP, MR-proANP and MR-proADM concentrations at baseline and delta values were analysed by linear regression analysis. In the multivariable models we included co-variables that were associated with the biomarkers in univariate analyses at the P <0.10 level.

All the models of predicted changes in biomarker levels after 52 week were analysed for the total cohort and for the intervention group. Since ∆total body weight and ∆fat mass were highly intercorrelated (r = 0.939; P < 0.001), only one of these parameters was included as a co-variable in each of the models. Accordingly, co-variables in models analysing ∆MR-proANP were: ∆fasting glucose, ∆total body weight or ∆fat mass. For MR-proADM: ∆ total body weight or ∆fat mass, ∆triglycerides (TG), ∆HDL-C, and ∆hs-CRP. No multivariable models were performed for ∆NT-proBNP. The biomarkers, NT-proBNP, MR-proANP and MR-proADM were logarithmically transformed (Ln) in the linear regression analyses to meet the assumption of linearity. Correlation coefficients (r) are shown for the univariable analyses, and the standardised coefficient (β) is shown for the multivariable linear regression analyses. All values are two tailed, and a P-value below 0.05 was considered statistically significant. The statistical software package SPSS version 20.0 was used for all analyses.

## Results

### Baseline analyses

A total of 52 obese individuals [47 women and 5 men; BMI (mean ± SD) 36.5 ± 5.6 kg/m^2^, and age 62 ± 7 yr] were investigated. Baseline characteristics according to randomisation group are presented in Table 1. The participants randomised to the diet intervention group were slightly younger and had lower MR-proANP concentrations than the control group. There were no differences in the remaining baseline parameters between groups.

**Table 1:** **Baseline characteristics according to randomization group**

	**Diet intervention (n = 28)**	**Controls (n = 24)**	**P value**
Age (yr)	59.5 ± 11.2	65.7 ± 9.6	0.038
Female/male (%)	93/7	88/12	0.51
*Body Composition*			
Body mass index (kg/m^2^)	37.1 ± 6.1	35.8 ± 5.1	0.41
Total body weight (kg)	97.5 ± 15.4	96.1 ± 16.3	0.75
Lean body mass (kg)	42.4 ± 8.7	43.4 ± 10.0	0.63
Fat mass (kg)	50.4 ± 12.6	48.3 ± 11.2	0.53
Fat (%)	51.5 ± 7.7	50.3 ± 7.3	0.59
*Metabolism*			
Cholesterol (mmol/l)	6.0 ± 1.1	5.8 ± 0.9	0.40
LDL-cholesterol (mmol/l)	3.7 ± 1.0	3.7 ± 0.8	0.94
HDL-cholesterol (mmol/l)	1.6 ± 0.4	1.5 ± 0.4	0.68
Triglycerides (mmol/l)	1.7 ± 1.3	1.2 ± 0.6	0.10
Fasting plasma glucose (mmol/l)	5.8 ± 0.7	5.9 ± 0.7	0.68
s-creatinine (μmol/l)	63 ± 12	65 ± 17	0.39
eGFR (ml/min)	94 ± 17	90 ± 21	0.42
*Biomarkers*			
NT-proBNP (pg/ml)	56 ± 30	41 ± 34	0.12
MR-proANP (pmol/l)	59 ± 21	77 ± 35	0.029
MR-proADM (pmol/l)	544 ± 152	604 ± 150	0.16
hs-CRP (mg/l)	5.5 ± 4.3	5.5 ± 4.2	0.99

Values are mean ± SD.

Linear regression analyses for the whole study population (n = 52) at baseline were performed to determine the impact of body composition on biomarker levels. We found no relationship between NT-proBNP, MR-proANP and BMI, but analysing body composition measurements demonstrated an inverse association between lean mass and NT-proBNP (r = −0.473; P < 0.001) and MR-proANP (r = −0.468; P < 0.001) levels.

We found gender modification with respect to both NPs, with the highest levels among women and adjustment for gender attenuated these correlations, being no longer statistically significant (P = 0.112) vs (P = 0.154) respectively, for NT-proBNP and MR-proANP. Further, TG was inversely correlated to both peptides, and serum creatinine correlated to MR-proANP. There was no association between fat mass and the NPs at baseline, and no association with glucose, other lipids or hs-CRP was observed. Regarding MR-proADM, there was no significant correlation with BMI (r = 0.211; P = 0.119) but, a trend towards a positive correlation with fat and with lean mass was noted (r = 0.249; P = 0.078 vs r = 0.260; P = 0.065, respectively). No association with lipids or plasma glucose was observed, MR-proADM being primarily correlated to serum creatinine (r = 0.312; P = 0.014).

### Analysis of changes during weight loss

The weight reduction programme had a marked effect for the intervention group; BMI decreased by almost 20% (from 37.1 ± 6.1 kg/m^2^ to 31.6 ± 6.2 P < 0.001) representing a mean weight loss of 15 ± 8%, and DEXA scans demonstrated a loss in body fat of 23.5 ± 15.5%; P < 0.001, while no significant decrease in lean mass was observed.

**Figure 1:**
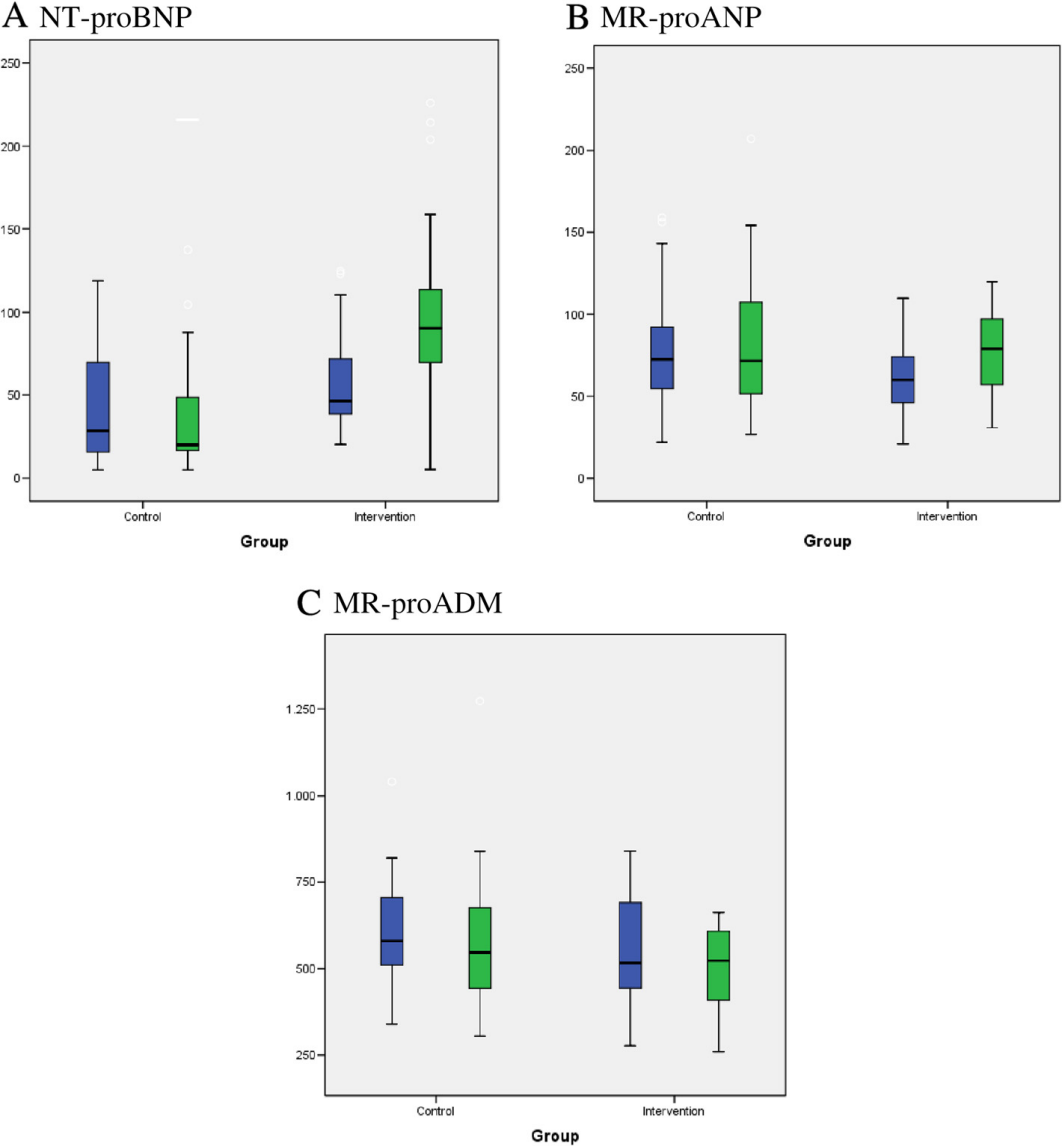
**A-C: Box-plots of levels of each biomarker according to randomisation group at baseline and after 1 year.** Boxes indicate the interquartile range and the crossbar the median. Changes between baseline and one yr follow-up in the intervention group: panel **A**: P<0.001, panel **B** and **C**: P<0.05. Changes between baseline and one yr follow-up in the control group, panel **C**: P<0.05.

Metabolic parameters associated with severe obesity such as plasma glucose and TG decreased during weight loss, while HDL-cholesterol increased. Plasma concentrations of the inflammatory biomarker hs-CRP were significantly reduced. The eGFR decreased from 130 to 116 ml/min (Table 2). After diet-induced weight loss, plasma NT-proBNP concentrations increased markedly from 55 ± 31 to 97 ± 55 pg/ml; P < 0.001, MR-proANP increased from 59 ± 21 to 74 ± 26 pmol/L; P < 0.001, and a decrease in MR-proADM levels from 573 ± 153 to 534 ± 173 pmol/L; P = 0.01 was observed. During the 52 weeks of observation, a modest but significant weight loss of 6% (P < 0.001) was found in the control group with no changes in plasma concentrations of NT-proBNP or MR-proANP, and a minor change in MR-proADM (Figure 1, Panel A-C). Since the MR-proANP levels differed between the two groups at baseline, percentage changes were analysed. In the intervention group MR-proANP increased by 16 ± 5%, while only an increase of 0.5 ± 5% was observed in the control group. The percentage increase in the intervention group was higher than in the control (P = 0.033). Delta changes in body composition measurements and in biomarker concentrations according to randomisation group are presented in Table 2. The mean changes in body weight, fat mass and in plasma NT-proBNP concentrations were significantly higher in the intervention group compared to the control group (P < 0.001).

**Table 2:** **Delta changes in baseline parameters after 52 weeks according to randomization group**

	**Intervention (n = 28)**	**Control (n =24)**	**Mean difference (95% CI)**	**P value (differences between groups)**
∆ Weight (kg)	−14.2 ± 1.6^a^	−6.1 ± 1.2^a^	−8.8 (−12.3, −3.9)	<0.0001
∆ BMI (kg/m^2^)	−5.4 ± 0.6^a^	−2.2 ± 0.3^a^	−3.2 (−4.8, −1.6)	<0.0001
∆ Fat mass (kg)	−11.5 ± 1.5^a^	−4.4 ± 1.2^a^	−7.0 (−11, −3.1)	0.001
∆ Fat-free mass (kg)	−0.9 ± 0.5^c^	−0.5 ± 4.0^c^	−0.4 (−1.7, 0.9)	0.54
∆ NT-proBNP (pg/ml)	42 ± 9^a^	4 ± 7^c^	−38 (−61, −14)	0.002
∆ MR-proANP (pmol/l)	15 ± 4^b^	5 ± 5^c^	−11 (−22, 1)	0.071
∆ MR-proADM (pmol/l)	−56 ± 15^b^	−19 ± 35^b^	37 (−37, 111)	0.32
∆ Plasma glucose (mmol/l)	−0.6 ± 0.3^a^	−0.4 ± 0.3^a^	−0.2 (−0.4, 0.2)	0.31
∆ CRP (mg/l)	−2.0 ± 0.9^b^	−0.9 ± 1.3^c^	−1.2 (−4.3, 2.0)	0.46
∆ Cholesterol (mmol/l)	−0.2 ± 0.2^c^	0.03 ± 0.2^c^	−0.3 (−0.6, 0.3)	0.50
∆ LDL cholesterol (mmol/l)	−0.2 ± 0.1^c^	−0.2 ± 0.1^c^	0.1 (−0.3, 0.5)	0.70
∆ HDL cholesterol (mmol/l)	0.1 ± 0.1^b^	0.2 ± 0.1^b^	0.1 (−0.1, 0.3)	0.22
∆ Triglycerides (mmol/l)	−0.5 ± 0.2^b^	0.05 ± 0.1^c^	−0.5 (−1.1, 0.1)	0.083
∆ s-creatinine (μmol/l)	−3.0 ± 1.0^b^	−5.0 ± 2.0^b^	1.8 (−3, 7)	0.45
∆ eGFR (ml/min)	6.1 ± 3.0^d^	7.0 ± 3.0^b^	1.0 (−8, 10)	0.82

Data are expressed as mean ± SEM, if not stated otherwise. NS, Not significant.

^a^P < 0.001, compared to baseline.

^b^P < 0.05, compared to baseline.

^c^no significant difference from baseline.

^d^P = 0.051.

### Predictors of change in biomarker concentrations

The impact of changes in weight, body composition and metabolic parameters on plasma concentrations of the three biomarkers were tested in a linear regression analysis for the pooled cohort of the intervention and the control group (n = 52) and for the intervention group (n = 28), evaluating delta predictors of change in MR-proANP, NT-proBNP and MR-proADM concentrations.

In the pooled cohort (n = 52), we found a univariable correlation between ∆MR-proANP and ∆total weight loss (r = −0.369; P = 0.008), ∆fat mass (r = −0.359; P = 0.011) and with ∆plasma glucose (r = −0.495; P < 0.001). Predictors of ∆MR-proANP were evaluated in a model only including ∆plasma glucose and ∆total weight or ∆fat mass, since correlation was high between ∆total weight loss and ∆fat mass (r = 0.939; P < 0.001). In this model, ∆fasting plasma glucose (β = −0.414; P = 0.005) was the only independent predictor of ∆MR-proANP, whereas changes in weight loss (P = 0.201) and in fat mass (P = 0.121) were no longer significant. Repeated analyses on the intervention group (n = 28) did not change this finding, with only ∆fasting plasma glucose (β = −0.446; P = 0.041) remaining a predictor of ∆MR-proANP. Finally, adjustment with ∆fat mass instead of ∆total weight did not change the β-coefficient of ∆glucose. We analysed predictors of ∆NT-proBNP for the total cohort, and found correlation with ∆plasma glucose (r = −0.462; P <0.001), whereas there was no significant association with ∆total weight (r = −0.207; P = 0.141) and with ∆fat mass (r = −0.208; P = 0.148). Further, no association was observed with ∆ lipid parameters. Evaluating predictors in the intervention group demonstrated a comparable association between ∆NT-proBNP and ∆plasma glucose (r = −0.455; P = 0.017), with no correlation with ∆fat mass and ∆ total weight.

With regard to ∆MR-proADM for the total cohort, univariable correlations with ∆total weight (r = 0.364; P = 0.009), with ∆fat mass (r = 0.330; P = 0.021), ∆ TG (r = 0.370; P = 0.026), ∆hs-CRP (r = 0.318; P = 0.013) and a trend with ∆HDL-cholesterol (r = −0.259; P = 0.067) were observed. Multivariable analyses demonstrated that ∆TG (β = 0.333; P = 0.035) and ∆hs-CRP (β = 0.361; P = 0.013) predicted the change in MR-proADM concentrations during weight loss, while the association with ∆total weight was no longer statistically significant (P = 0.176). However, entering ∆fat mass in this model revealed that the decrease in total fat mass was a stronger and independent predictor of changes in MR-proADM levels at 52 weeks (β = 0.330; P = 0.021). There were no association between changes in eGFR and changes in neither NPs nor MRpro-ADM concentrations.

For the intervention group, we found that the reduction in total body weight was predictive of ∆MR-proADM (β = 0.397; P = 0.027), and in a model with ∆fat mass a trend towards an association was found (β = 0.333; P = 0.060) after adjustment for the same co-variables (∆TG, ∆hs-CRP and ∆HDL-cholesterol).

## Discussion

We report that in obese subjects without heart disease, plasma levels of both NT-proBNP and MR-proANP increase after substantial weight loss on a very low calorie diet. To the best of our knowledge, the present study is the first to examine the impact of weight loss and changes in body composition on plasma concentrations of MR-proANP and NT-proBNP and MR-proADM in a randomised controlled intervention study. The increase in MR-proANP was associated with reduction of fat mass and fasting plasma glucose, whereas for NT-proBNP primarily a relationship with the improvement of plasma glucose was observed. Plasma MR-proADM concentrations decreased, and our data suggest a relationship with reduced body weight, body fat, and with plasma hs-CRP levels.

Previous studies of changes in natriuretic peptides due to weight loss have been observational, predominantly reporting the effects of bariatric surgery on B-type NPs, and with conflicting findings [7,8,19-21]. Two longitudinal studies, which measured NT-proBNP before and after bariatric surgery observed similar increases in plasma concentrations six months after surgery [20,21]. Accordingly, a retrospective study observed elevated BNP and NT-proBNP concentration in obese patients with a history of gastric bypass surgery [7]. In contrast, Hanusch-Enserer et al. [8] found elevated NT-proBNP plasma levels in obese subjects which decreased 12 months after surgery, using an in-house assay for measurements of NT-proBNP. With respect to the impact of dietary intervention, the available data is sparse and conflicting. Chainani We et al. [22] reported an increase in BNP after weight loss due to lifestyle changes in a high risk population, whereas a small study on 12 obese subjects observed a decrease in BNP and ANP levels [23].

An independent effect of bariatric surgery has been suggested in a recent study, reporting a six-fold increase in NT-proBNP levels two days after surgery, with a subsequent decrease over the next four days. In addition, they observed an increase in NT-proBNP from 53 to 122 pg/ml during the first post-operative year, [19] which is in line with our current findings. This indicates an effect of fluid load during the first post-operative days however, a potential hormonal influence of gastric bypass surgery can not been ruled out. Therefore the majority of the available data is in accordance with the present findings of an approximately two-fold rise in NT-proBNP concentrations after substantial weight loss of 15–20% body weight.

The mechanisms controlling decreased levels of circulating NPs in obese subjects are not known. It has been hypothesised that obesity mediates an up-regulation of the natriuretic peptide clearance receptor type-C (NPR-C) in adipose tissue, which consequently increases peripheral elimination from the circulation. Conversely, the current findings that both NT-proBNP and MR-proANP, which are not cleared by NPR-C, increase after loss of body fat suggest that production of natriuretic peptides from cardiac myocytes could be impaired in obese individuals. Accordingly, recent experimental data from our group has demonstrated decreased expression of both the ANP and BNP gene in the cardiac ventricles and lipid accumulation in the myocytes of obese mice [24].

The NPs have been recognised as important regulators of fat metabolism. They can stimulate lipolysis in humans [25], and recently NPs have been demonstrated to promote increased energy expenditure by browning of adipocytes [26]. Thus, impaired synthesis of cardiac NPs in obese subjects could be related to their accumulation of fat.

We evaluated predictors of change in NT-proBNP, MR-proANP and MR-proADM concentrations using DEXA scanning to measure fat and muscle mass. Our results suggest that loss of body fat is associated with an increase in MR-proANP concentrations, whereas only a trend toward an association with changes in NT-proBNP was found. It is worth noting that we did not observe an association between baseline total fat mass and NT-proBNP or MR-proANP in our cohort. However, using whole body DEXA scanning rather than measuring the biologically active abdominal fat could be of implication, since a significant relation between visceral abdominal fat and plasma BNP levels has been observed [27,28]. The impact of weight loss is primarily reducing abdominal fat, which could in part explain the relatively weak relationship between the changes in fat mass and plasma concentrations of natriuretic peptides in our study.

At the baseline, plasma NT-proBNP concentrations were not associated with BMI, which has been the general finding in population-based cohorts and in heart failure [1,29]. However, an inverse association with lean mass was observed, and it has previously been suggested that B-type natriuretic peptides may be more strongly correlated with lean mass [30]. We report the novel finding of a similar inverse association between high lean mass and decreased MR-proANP concentrations. The association was similar for NT-proBNP and MR-proANP, therefore a common non-receptor mediated clearance mechanism may be partly related to reduced concentrations of natriuretic peptides in obesity. Accordingly, NT-proBNP may be cleared from multiple tissues including muscle [31]. Furthermore, a possible role for androgen hormones has been suggested, hence free testosterone is inversely associated with plasma BNP and NT-proBNP concentrations in young women, and androgens may inhibit natriuretic peptide release from myocytes [32]. The present data supports an impact of androgen hormones, as we could not demonstrate an association between the NPs and lean mass after adjustment for gender. This is further supported in a recent report from the Dallas Heart Study, since measurements of free testosterone modified the effect of lean mass on BNP and NT-proBNP, being non-significantly associated after adjustment for testosterone levels [33]. However, we could not demonstrate an association between lean mass and NPs during follow-up. The mechanisms behind this finding are speculative, but the relatively modest loss of muscle tissue in our study could be of significance. The present low calorie and relatively high protein diet in the intervention group could play a role with regards to the very low loss of FFM [34,35]. In addition, methodologically considerations should be noted, since FFM measured by DEXA can be affected by changes in hydration status over time.

The present study suggests a possible interaction between glucose metabolism and natriuretic peptides, since a reduction in fasting plasma glucose was an independent determinant of the increase in NT-proBNP and MR-proANP concentrations. The potential mechanism for this has not been investigated in detail. However, of interest it has recently been reported that BNP infusion reduces plasma glucose levels in healthy males, thus supporting a possible relationship between the A and B type natriuretic peptides and glucose homeostasis [36]. We did not find any significant association between changes in TG levels and NPs, which could be suspected given the proposed relation with lipid metabolism. Future studies including more detailed measurements of lipid and glucose metabolism should address this issue.

The current finding of a decrease in MR-proADM concentrations during weight loss is in accordance with one previous observational study among patients undergoing gastric bypass surgery. They also reported a positive correlation with BMI in 350 obese individuals [14]. We were not able to demonstrate a statistically significant relationship at the baseline, but the relatively small sample size of our study may account for this discrepancy. We examined body composition and found a trend towards a positive correlation between MR-proADM and fat and lean tissue mass. To our knowledge, the relationship with fat and lean tissue mass has not previously been studied and may require further investigation. During weight loss the decrease in MR-proADM correlated with a reduction in fat mass and TG, indicating a reduced release into the circulation from adipocytes [9]. In addition, the decrease in hs-CRP levels during weight loss was an independent determinant of changes in plasma MR-proADM concentrations, which is of potential interest and in accordance with findings in type 2 diabetic patients [13]. Inflammatory cytokines act as stimulatory factors of ADM production, and could thus be of clinical significance [37]. Finally, we could not find any relationship between the decrease in MR-proADM and change in eGFR, probably reflecting the modest and not clinically significant decrease in eGFR observed in our cohort.

The strengths of the current study include the magnitude of weight loss achieved by dietary intervention, use of DEXA, measurements of both A and B-type natriuretic peptides as well as adrenomedullin and the prospective design and use of a relatively well matched control group. The randomised nature of our study is an advantage, but the two groups were not identical with respect to age and MR-proANP levels at the baseline. There is no apparent explanation for this discrepancy, which we believe has occurred by chance, and it could be related to the limited number of participants in our study.

There are several limitations to our study that should be addressed. Thus, it would be of interest in future clinical investigations to include measurements of visceral and subcutaneous fat, in order to assess whether reduction of metabolically active abdominal fat tissue has an impact on circulating A and B-type natriuretic peptides. Unfortunately, these measurements were not available in our present study. In addition, we included primarily women, which are a general issue in obesity studies, consequently only having data on n = 5 males should be considered when interpreting data regarding gender differences.

Measurement of left ventricular function using echocardiography was not conducted, which could be argued as a potential limitation, being the main source of natriuretic peptide secretion. However, we do not consider the increase in circulating NT-proBNP and MR-proANP to be mediated by cardiac impairment, since improved function of the left ventricle has been reported two years after substantial weight loss following gastric bypass [38]. In addition, data on history of hypertension was not available which should be considered as a potential limitation. At the time of randomisation none of the subjects had any symptoms related to heart disease, and further, NT-proBNP levels were below the recommended cut-off value for heart failure. Baseline levels of MR-proANP differed between groups at baseline, which also should be considered as a limitation. Finally, since plasma glucose was independently predictive of changes in MR-proANP and NT-proBNP suggesting a relationship with glucose metabolism, measurements of plasma insulin would be of interest in future studies. This is further supported by data suggesting that insulin may suppress circulating NP concentration by inducing an up-regulation of the NPRC expression in adipocytes in obese subjects [39].

## Conclusions

- Plasma concentrations of NT-proBNP and MR-proANP increase significantly during diet-induced substantial weight loss.
- BMI and weight changes should be considered when interpreting NT- proBNP and MR-proANP levels.
- The main predictors of changes in NPs during weight loss are the reduction in fat mass and improvement in plasma glucose levels.
